# Telomere length dynamics over 10-years and related outcomes in patients with COPD

**DOI:** 10.1186/s12931-021-01616-z

**Published:** 2021-02-15

**Authors:** E. Córdoba-Lanús, S. Cazorla-Rivero, M. A. García-Bello, D. Mayato, F. Gonzalvo, J. Ayra-Plasencia, B. Celli, C. Casanova

**Affiliations:** 1 Research Unit, Hospital Universitario Nuestra Señora de Candelaria, Santa Cruz de Tenerife, Spain; 2 Pulmonary Division, Hospital Universitario Nuestra Señora de Candelaria, Santa Cruz de Tenerife, Spain; 3 University of La Laguna, San Cristóbal de La Laguna, Tenerife, Spain; 4 Instituto Universitario de Enfermedades Tropicales Y Salud Pública de Canarias (IUETSPC), Tenerife, Spain; 5 Pulmonary and Critical Care Department, Brigham and Women’s Hospital, Boston, MA USA

**Keywords:** Aging, COPD, Lung-function, Mortality, Telomeres

## Abstract

**Background:**

Chronic obstructive pulmonary disease (COPD) has been proposed as a disease of accelerated aging. Several cross-sectional studies have related a shorter telomere length (TL), a marker of biological aging, with COPD outcomes. Whether accelerated telomere shortening over time relates to worse outcomes in COPD patients, is not known.

**Methods:**

Relative telomere length (T/S) was determined by qPCR in DNA samples from peripheral blood in 263 patients at baseline and up to 10 years post enrolment. Yearly clinical and lung function data of 134 patients with at least two-time measures of T/S over this time were included in the analysis.

**Results:**

At baseline, T/S inversely correlated with age (r = − 0.236; p < 0.001), but there was no relationship between T/S and clinical and lung function variables (p > 0.05). Over 10 years of observation, there was a median shortening of TL of 183 bp/year for COPD patients. After adjusting for age, gender, active smoking and mean T/S, patients that shortened their telomeres the most over time, had worse gas exchange, more lung hyperinflation and extrapulmonary affection during the follow-up, (PaO_2_ p < 0.0001; K_CO_ p = 0.042; IC/TLC p < 0.0001; 6MWD p = 0.004 and BODE index p = 0.009). Patients in the lowest tertile of T/S through the follow-up period had an increased risk of death [HR = 5.48, (1.23–24.42) p = 0.026].

**Conclusions:**

This prospective study shows an association between accelerated telomere shortening and progressive worsening of pulmonary gas exchange, lung hyperinflation and extrapulmonary affection in COPD patients. Moreover, persistently shorter telomeres over this observation time increase the risk for all-cause mortality.

## Background

Chronic obstructive pulmonary disease (COPD), one of the leading causes of morbidity and mortality worldwide, is a disease characterized by a persistent reduction of airflow that frequently progresses over time [1, 2]. In addition, patients with COPD develop 10 or 20 years earlier, comorbid diseases characteristically seen in elderly subjects without COPD [3, 4].

COPD has been described as a disease of accelerated aging and shorter telomere length as a surrogate marker of biological aging [5, 6]. In humans, telomeres consist of a repeating sequence of TAAGGG hexanucleotide located at the ends of chromosomes and have an important role in maintaining chromosome integrity and cell proliferation [7]. Telomeres shorten 30–100 base pairs during each cell division due to the end-replication problem of the DNA polymerase [8, 9]. Telomere shortening and telomere dysfunction may heavily influence the aging human lung [10]. It has been shown that patients with COPD exhibit shorter leucocyte telomeres when compared with smokers without COPD and healthy subjects [11–13]. Importantly, we have also shown that COPD patients experience accelerated telomere shortening over time when compared to smoking controls [13].

Shortening of telomere length may be a risk factor for all-cause or cause-specific mortality [14, 15]. The same appears to be true for patients with COPD, as shorter telomere length has been associated with worse lung function [16, 17], exacerbations and risk of death [18, 19]. However, these studies suggesting an association between telomere length and respiratory health were cross sectional in design. There is one recent study in the general population relating telomere length with longitudinal assessment of clinical data, but the study had only one measure of telomere length at baseline. In that study, smokers with short telomeres at baseline had accelerated lung function decline over time [20]. No long-term study of patients with COPD, has measured telomere length over time and explored the association between changes in telomere length and clinical and physiological variables of importance to those patients.

The aim of the present study was to test the hypothesis that telomere length shortening over time in patients with COPD is associated to clinical, lung function, and patient-related outcomes in 10 years of follow-up.

## Methods

### Subjects

**Fig. 1:**
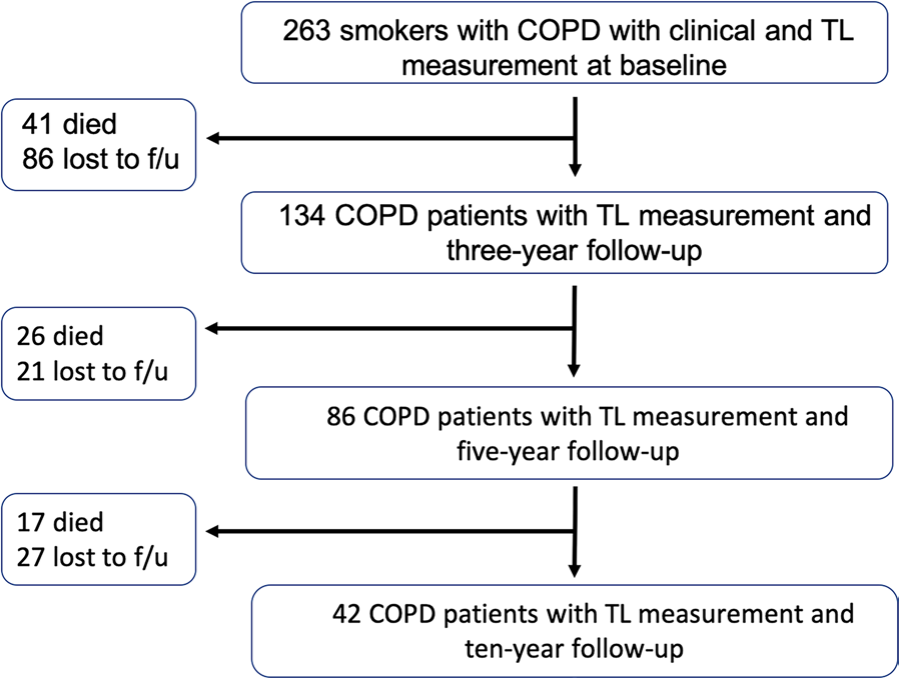
Flow diagram representing individuals recruited for the present study

A total of 263 were smokers with COPD diagnosis were screened for this study at the Hospital Universitario La Candelaria, Tenerife, Spain (Tenerife-cohort) that were followed annually as part of the BODE cohort [21, 22]. Inclusion criteria: age > 40 years, smoking history > 15 pack-years and post-bronchodilator FEV_1_/FVC ratio < 0.70 clinically stable for at least 6 weeks at the time of evaluation. Spirometry, lung volumes and exercise capacity were measured according to ATS-ERS guidelines [23, 24]. Dyspnea, evaluated by mMRC scale [25], BODE Index [21] and Charlson index for comorbidities [26] were registered at every visit. Exclusion criteria: uncontrolled co-morbidities such as malignancy at baseline, asthma or other pulmonary conditions than COPD. Exacerbations were defined as a worsening of respiratory symptoms (dyspnea, cough or sputum) that required the use of antibiotics, systemic corticosteroids, or both or necessitated emergency room visit or hospital admission. All-cause mortality was recorded using information obtained from the family and then confirmed by reviewing medical records (Fig. 1).

Longitudinal study: included 134 patients from the overall cohort that were monitored through the 10 years of follow-up (413 observations). These patients presented at least two-time longitudinal measures of telomere length and a mean follow-up of 6 years. From these 42 reached 10 years of observation. As show in Additional file 1: Table S1, the clinical characteristics of these 42 patients were similar to that of the cohort as a whole, except for being slightly younger, presenting higher K_CO_ and able to walk more on the 6MWD test.

In each annual visit of the recruited participants peripheral blood sample was taken and all the clinical and functional parameters were recorded.

The study was approved by the institutional review board of HUNSC (PI14/12). All participants provided written informed consent.

### Telomere length measurement

DNA was extracted from whole blood obtained at baseline, the 3rd-year, the 5th-year and at the 10th-year post enrolment. The QIAamp DNA Mini Kit (GE Healthcare) was used for this purpose and the resulting DNA samples were quantified using the Nanodrop lite spectrophotometer (Thermo Scientific, Wilmington, DE, USA). Telomere length was measured in triplicate in each sample (20 ng of DNA) using a qPCR-based protocol as described in a previous publication of our group [13]. Also, calibrator samples were assayed in triplicate on each PCR plate to control for variation between plates. Intra-plate coefficients of variance (CV) were calculated between the replicates and samples with CV > 5% were excluded from further analysis. Two control DNA samples were assay per run as a normalizing factor. Inter-plate CV for the calibrator sample was calculated to be < 8.5%. Albumin, a single copy gene, was used as a reference gene.

Telomere length was calculated as a ratio of telomere to albumin where the T/S ratio for an experimental DNA sample is T, the number of nanograms of the standard DNA that matches the experimental sample for copy number of the telomere template, divided by S, the number of nanograms of the standard DNA that matches the experimental sample for copy number of the albumin single copy gene [27]. T/S was calculated using the “∆∆Cp with efficiency correction” calculation method [28].

### TRF southern blot analysis

Telomere restriction fragment analysis [29] was performed by southern blot using the TeloTAGGG Telomere Length Assay Kit (Roche) according to the manufacturer instructions. The mean telomere length was calculated using the following: TRF = ∑(ODi)/∑(ODi/Li), where ODi is the chemiluminescent signal and ODi/Li is the length of the TRF at position. Conversion of T/S ratio to base pair was calculated for every subject based on the equation: y = 1114.58 + 10,373.13 * x of the correlation analysis, where x is T/S ratio (Additional file 2: Figure S1).

### Statistical analysis

#### Baseline characteristics and outcomes

The 263 patients with COPD were categorized in three groups by relative telomere length ratio (T/S) tertiles at baseline: shorter, medium or longer telomeres. T/S was inversely correlated with age, so all subsequent analyses were adjusted by this variable. Differences in means and proportions of baseline and follow-up characteristics between groups of patients were tested using t-Student, ANOVA, Chi^2^, Fisher Exact, Kruskal–Wallis.

#### Telomere length shortening over time and outcomes

Longitudinal analysis was performed on each individual having at least two-time T/S measures during their follow-up over 10 years. A total of 134 patients were evaluated during follow-up until time of censoring (drop-out or death). In this analysis, we considered mortality as events that occurred during follow-up within the 3 years after the last clinical evaluation (n = 43). A linear regression mixed model for repeated measures was performed to test the association of telomere length dynamics over follow-up time and the clinical and pulmonary function variables. The effect of the change in relative telomere length through time was analysed in relation to pulmonary function variables measured during the observation time by the variable T/S_mCh: as the change in T/S with respect to its mean over time in each individual. The age, gender and the mean T/S of each individual were used as covariates.

To analyse the effect of telomere length on all-cause mortality we compared the risk of mortality across T/S in each individual of the entire cohort over the total follow-up period by using a Cox proportional hazards ratio (HR) regression model in multivariate analysis. T/S was measured four times; at baseline, at the third, the 5th and at the 10th-year post enrolment. Because every subject included had an annual evaluation of their clinical and lung function parameters, we used the last observation carried forward (LOCF) approach to manage the T/S measures registered at the four moments (baseline, the 3rd-year, the 5th-year and at the 10th-year post enrolment). Individual T/S values were analysed using the last observation (T/S) registered, and carried forward in order to construct the different models.

Mortality risk was tested in every subject included in the study throughout its follow-up and over the subsequent 12 months from the last clinical evaluation, taking into account that they had at least two-time longitudinal T/S measures. Kaplan–Meier estimator is used to illustrate the association between this time varying covariate and mortality as a clinical outcome. In the multivariate model, the following covariates were included: age, gender, smoking status (pack-years of smoking), active smoking (current or ex-smokers), FEV_1_%, BODE index and 6MWD every year of follow-up.

SPSS 25.0 IBM Co and R software were used for all statistical analyses and two-tailed p-values < 0.05 were considered significant.

## Results

### Baseline analysis

The clinical characteristics and lung function data of 263 COPD patients at baseline distributed by tertiles of relative telomere length are summarized in Table 1. The range of airflow obstruction distributed by GOLD stages in COPD was as follows: I (16.7%), II (43%), III (30.8%) and IV (9.5%). Individuals with shorter telomeres were older and had a higher number of pack-years smoked (p = 0.023). There was no relationship between telomere length and clinical and lung function parameters (p > 0.05) cross-sectionally. Telomere length measured by the T/S ratio inversely correlated with age (r = − 0.236; p < 0.001). The median TL of the patients` with an average of 64 years old, was 7.8 ± 2.7 kbp. Additional file 2: Figure S1 shows that on average, telomere length was shorter as age increased.

**Table 1:** Baseline clinical and lung function characteristics of COPD patients grouped by relative telomere length tertiles

Variable	Short T/S^d^ N = 87	Medium T/S^d^ N = 88	Long T/S^d^ N = 88	p-value
T/S ratio^a^	0.40 ± 0.08	0.60 ± 0.05	0.92 ± 0.22	*< 0.001*
TL (bp)^a^	5248 ± 855	7346 ± 535	10,757 ± 2371	*< 0.001*
Age^a^	66 ± 9	64 ± 9	61 ± 10	*0.005*
Sex (male %)	76	80	66	0.104
BMI^a^	28 ± 6	28 ± 5	26 ± 5	0.068
Smoking habit (pack-year)^a,c^	69 ± 30	65 ± 26	58 ± 24	*0.023*
Active smoking (%)	43	43	40	0.888
FEV_1_ (L)^a^	1.49 ± 0.64	1.60 ± 0.65	1.46 ± 0.66	0.326
FEV_1_ (% pred)^a^	58 ± 21	59 ± 20	55 ± 23	0.544
FVC (% pred)^a^	87 ± 21	90 ± 23	85 ± 23	0.348
FEV_1_/FVC (% pred)^a^	52 ± 13	51 ± 11	51 ± 13	0.930
PaO_2_^a^	71 ± 12	73 ± 11	71 ± 12	0.605
K_CO_^a^	79 ± 27	77 ± 24	69 ± 26	0.059
ICTLC (%)^a^	35 ± 8	35 ± 8	34 ± 10	0.875
6MWD (mts)^a^	477 ± 94	486 ± 102	480 ± 105	0.824
mMRC dysnea^b^	1 (0–2)	1 (0–2)	1 (0–2)	0.210
BODE index^b^	1 (0–2)	1 (0–3)	1 (0–3)	0.223
Charlson index^b^	0 (0–1)	0 (0–1)	0 (0–1)	0.769
Exacerbations^b^	0 (0–1)	1 (0–2)	0 (0–1)	0.146

*T/S ratio* relative telomere length, *TL* telomere length, *BMI* body mass index, *FEV*_*1*_ forced expiratory volume in 1 s, *FVC* forced vital capacity, *% pred* per cent predicted, *PaO*_*2*_ partial oxygen tension, *K*_*CO*_ transfer factor coefficient of the lung for carbon monoxide, which is DL_CO_, *IC/TLC* inspiratory capacity to total lung capacity ratio, *6MWD* 6 min walking distance test.* p*-values < 0.05 are shown in italics

^a^Data are presented as mean ± SD

^b^Data are presented as median (25th–75th pc)

^c^Number of packs of cigarettes smoked per day × number of years smoking

^d^Groups defined by relative telomere length (T/S) tertiles: < 0.52, 0.52–0.71 and > 0.71

### TFR by southern blot analysis

Telomere length was measured in forty COPD patients’ DNA samples using southern blot. Relative telomere length (T/S) measured by qPCR in these same samples correlated with telomere length TL in base pairs measured by southern blot (r = 0.502, p = 0.001) (Additional file 3: Figure S2).

### Longitudinal analysis

The longitudinal study was performed in the 134 patients followed annually over 10 years that presented at least two T/S measures over that time. The clinical and pulmonary function characteristics of these patients are shown in Table 2.

**Table 2:** Baseline characterization of COPD patients who achieved 10 years of follow-up and those who died

Variable	Total patients included (n = 134)	Alive after 10 year-follow-up (n = 42)^‡^	Died during 10 year-follow-up (n = 43)^‡^	p-value^‡^
Age	64 9	61 ± 8	69 ± 9	*0.001*
BMI^a^	27 ± 5	27 ± 6	28 ± 5	0.781
Smoking habit (pack-year)^a,c^	65 ± 26	61 ± 22	70 ± 31	0.139
Active smoking (%)	43	52	33	0.069
FEV_1_ (L)^a^	1.52 ± 0.62	1.61 ± 0.61	1.34 ± 0.56	*0.038*
FEV_1_ (% pred)^a^	58 ± 21	61 ± 19	51 ± 21	*0.027*
FVC (% pred)^a^	89 ± 24	90 ± 23	81 ± 25	*0.070*
FEV_1_/FVC (% pred)^a^	51 ± 11	54 ± 10	49 ± 11	*0.024*
PaO_2_^a^	72 ± 11	73 ± 10	68 ± 10	*0.015*
K_CO_^a^	80 ± 26	90 ± 28	74 ± 23	*0.009*
IC/TLC (%)^a^	34 ± 8	35 ± 8	33 ± 8	0.143
6MWD (mts)^a^	495 ± 90	522 ± 83	474 ± 75	*0.008*
mMRC dysnea^b^	1 (0–2)	1 (0–1)	1 (0–2)	0.703
BODE index^b^	1 (0–3)	1 (0–2)	1 (0–3)	0.179
Charlson index^b^	0 (0–1)	0 (0–1)	1 (0–1)	0.740

Paired sample t test was used

*BMI* body mass index, *FEV*_*1*_ forced expiratory volume in 1 s, *FVC* forced vital capacity, *% pred* per cent predicted, *PaO*_*2*_ partial oxygen tension, *K*_*CO*_ transfer factor coefficient of the lung for carbon monoxide, which is DL_CO_, *IC/TLC* inspiratory capacity to total lung capacity ratio, *SMWD* 6 min walking distance test

^‡^p-value between compared groups

^a^Data are presented as mean ± SD

^b^Data are presented as median (25th–75th pc)

^c^Number of packs of cigarettes smoked per day × number of years smoking

Table 3 shows the clinical and pulmonary function characteristics at baseline and after 10 years of follow up in the 42 patients that completed that period of observation. They were mostly men (67%) and had a medium age of 61 ± 8 at baseline. The telomere length was 7583 ± 2328 bp when first recruited and 5755 ± 1456 bp 10 years later. The medium loss in TL observed was 183 bp/year.

**Table 3:** Characterization of patients with COPD (N = 42) at baseline and at 10 years of follow-up

Variable	Baseline	10 year-follow-up	p-value
T/S ratio^a^	0.62 ± 0.22	0.45 ± 0.14	*< 0.0001*
TL (bp)^a^	7583 ± 2328	5755 ± 1455	*< 0.0001*
BMI^a^	27 ± 6	27 ± 6	0.981
Active smoking (%)	52	40	*< 0.0001*
FEV_1_ (L)^a^	1.61 ± 0.61	1.36 ± 0.57	*< 0.0001*
FEV_1_ (% pred)^a^	61 ± 19	56 ± 18	*0.014*
FVC (% pred)^a^	90 ± 23	86 ± 24	*0.043*
FEV_1_/FVC (% pred)^a^	54 ± 10	51 ± 10	*0.022*
PaO_2_^a^	73 ± 10	68 ± 10	*< 0.0001*
K_CO_^a^	90 ± 28	76 ± 24	*0.006*
IC/TLC (%)^a^	36 ± 8	31 ± 9	*0.001*
6MWD (mts)^a^	520 ± 84	448 ± 125	*< 0.0001*
mMRC dysnea^b^	1 (0–1)	1 (0–2)	0.209
BODE index^b^	1 (0–2)	1 (0–3)	*0.003*
Charlson index^b^	0 (0–1)	1 (0–1)	*< 0.0001*

Paired sample t test was used

*T/S ratio* relative telomere length, *TL* telomere length in base pairs, *BMI* body mass index, *FEV*_*1*_ forced expiratory volume in 1 s, *FVC* forced vital capacity, *% pred* per cent predicted, *PaO*_*2*_ partial oxygen tension, *K*_*CO*_ transfer factor coefficient of the lung for carbon monoxide, which is DL_CO_, *IC/TLC* inspiratory capacity to total lung capacity ratio, *SMWD* 6 min walking distance test.* p*-values < 0.05 are shown in italics

^a^Data are presented as mean ± SD

^b^Data are presented as median (25th–75th pc)

### Telomere length shortening and pulmonary function

The effect of the change in T/S in relation to its mean value was analysed in each of the 134 patients included in the longitudinal study throughout their follow-up period. Overall patients that shortened the most their telomeres over that time, had worse pulmonary gas exchange measure by PaO_2_, K_CO_, worse static lung hyperinflation (IC/TLC) and extrapulmonary affection (BODE index), even after adjustment by age, gender, active smoking and the mean T/S of each subject (Table 4). Moreover, patients that died during the follow-up period had more telomere shortening in relation to the same clinical and pulmonary function variables.

**Table 4:** Longitudinal association between decreased telomere length and lung function and clinical variables during 10 year-follow-up

	Total patients (n = 134)		Deaths during follow-up (n = 43)		Alive patients (n = 91)	
	β	p-value	β	p-value	β	p-value
FEV_1_ (L)	0.13	*0.022*	0.17	0.112	0.11	0.104
FEV_1_ (% pred)	0.56	0.784	2.91	0.501	0.59	0.798
FEV_1_/FVC (% pred)	0.445	*0.0008*	0.579	*0.024*	0.377	*0.015*
PaO_2_	0.771	*< 0.0001*	1.398	*0.0003*	0.443	*0.053*
IC/TLC (%)	0.006	*< 0.0001*	0.006	*0.021*	0.006	*0.0009*
K_CO_	0.877	*0.042*	1.325	0.092	0.817	0.106
6MWD (mts)	4.655	*0.004*	10.53	*0.007*	1.769	0.299
BODE index	− 0.081	*0.009*	− 0.219	*0.002*	− 0.013	0.689

Linear regression of mixed models. β, coefficient

*FEV*_*1*_ forced expiratory volume in 1 s, *FVC* forced vital capacity, *% pred* per cent predicted, *PaO*_*2*_ partial oxygen tension, *K*_*CO*_ transfer factor coefficient of the lung for carbon monoxide, which is DL_CO_, *IC/TLC* inspiratory capacity to total lung capacity ratio, *6MWD* 6 min walking distance test.* p*-values < 0.05 are shown in italics

### Telomere length and mortality risk

**Fig. 2:**
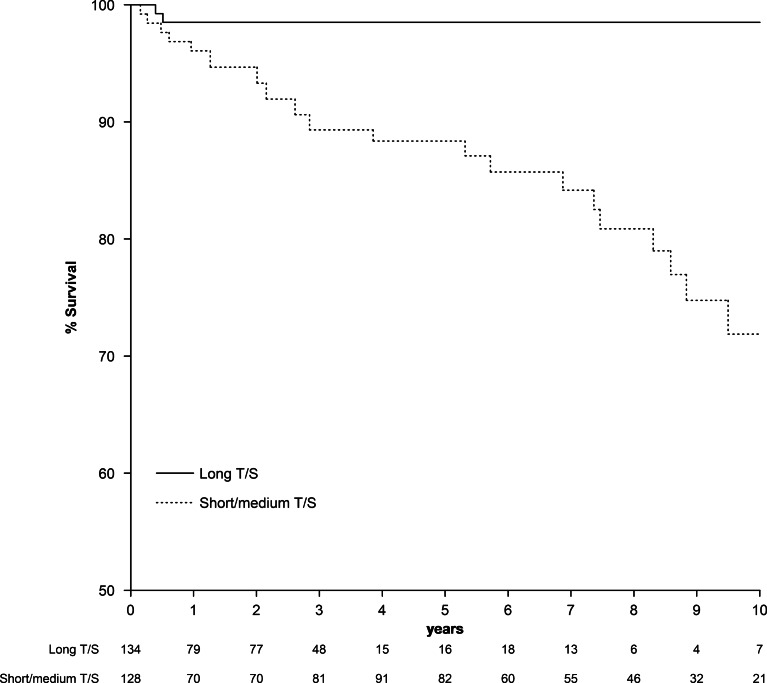
Kaplan–Meier survival curves for COPD patients with different telomere length. Patients were divided into tertiles of T/S (relative telomere length ratio): T1 (long T/S), T2 (medium T/S) and T3 (short T/S)

During the follow-up period, 87 (33%) of the participants died (19.5% from cancer, 39.1% from a respiratory cause and 5.8% from a cardiovascular cause). Patients with COPD with shorter telomeres (T1 and T2 tertiles of T/S) showed a higher risk of all-cause mortality (Cox HR = 5.481, p = 0.026) (Table 5, Fig. 2). In the overall cohort, the individual variation of a decrement in 0.1 units of T/S over time increased the risk of mortality (HR = 1.446, p = 0.009).

**Table 5:** Hazard ratio of all-cause mortality in patients with COPD grouped by tertiles of telomere length

	HR (95% Cl)	p-value
Model 1		
Medium vs. long T/S^a^	4.803 (0.99–23.18)	0.051
Short vs. long T/S^a^	6.267 (1.32–29.82)	*0.021*
Model 2		
Short/medium vs. long T/S^a^	5.481 (1.23–24.42)	*0.026*
Model 3		
T/S (decrement 0.1 units)^a^	1.446 (1.10–1.91)	*0.009*

*T/S* relative telomere length ratio, *CI* confidence interval

^a^Cox HR analysis was adjusted by age, FEV_1_% and active smoking (current and former smokers) as covariates. Long T/S was used as the reference level.* p*-value < 0.05 are shown in italics

## Discussion

To our knowledge this is the first study to explore the relationship between telomere length change over 10 years and clinical outcomes, in a cohort of COPD patients. Those patients that shorten their telomeres the most during the follow-up period, showed worsening of alveolar gas exchange, lung hyperinflation and clinical outcomes compared with those whose telomeres did not shorten as much. Moreover, patients within the lowest telomere length presented a higher risk of all-cause mortality.

According to previous studies completed in general populations, leucocyte telomeres shorten 40–105 base pairs per year [14, 30]. We found that the mean telomere length in this cohort of COPD patients aged 64 years-old at time of recruitment was 7.6 kbp and it decreased to 5.7 kbp after 10 years, approximately 183 bp/year. In addition, the TL observed in COPD patients in this study corresponds to that observed by others in healthy subjects of similar age but 10 years older [14]. Rutten and colleagues also suggested an anticipated telomere attrition in patients with COPD corresponding to a biological age 7 years older [17]. Other studies using clinical observations but without telomere length determination, have suggested a relationship between COPD severity, and the development of diseases characteristically seen in the elderly [31–33]. Recently, Divo and co-workers [4] using comorbidities network analysis showed that patients with COPD developed a similar prevalence of diseases frequently seen in the elderly one or two decades earlier than in patients without COPD. These observations supporting accelerated aging as a potential mechanism in patients with COPD, should be associated with accelerated shortening of telomeres if this were a marker of aging. Indeed, we have previously shown that COPD patients shorten their telomeres over time at a higher rate than healthy individuals of the same age [13].

In this prospective study, COPD patients that shortened their telomeres the most over the 10 years of observation had significantly worse oxygenation (PaO_2_), lower K_CO,_ more hyperinflation (IC/TLC), lower BODE index and lower 6MWT than those patients with less telomere shortening. As shown in Table 3, these associations were stronger than that observed between telomere shortening and the change in FEV_1_% predicted, suggesting that accelerated aging affects primarily the lung parenchyma over the airway tissue itself. In addition, the effects also seem to impact intensely in the extra-pulmonary components of the disease. In this way, patients with shorter telomeres over time score worse the BODE index, a good predictor of poor outcomes, compared to the FEV_1_. Previously, one other study reported a correlation between shorter telomere length and worse oxygenation but not with lung function expressed by the FEV_1_ [11]. Moreover, other authors proposed that telomere attrition may act as biomarkers of COPD severity [17, 34], impaired exercise capacity [35], health status (activity score domain of the SGRQ) and exacerbations [19, 35]. We expand on these findings by demonstrating for the first time a relationship between accelerated telomere shortening and worsening of alveolar gas exchange and clinical extrapulmonary variables in patients with COPD, thus supporting the concept that telomere shortening is a surrogate marker of the aging process in vivo [36].

Interestingly, individuals in the lowest tertile of telomere length through the follow-up were at an increased risk of mortality when compared to the highest tertile of TL, independent of age, active smoking and lung function. A telomere length ratio decrement of 0.1 units had a predictive risk value for all-cause mortality. Contradictory results have been reported from studies on general population exploring the relation between TL and mortality [15, 37, 38], but very few studies have been completed in patients with COPD. Our findings are in agreement with Lee and co-workers [18] who found that leucocyte telomere length was related to all-cause and cancer mortality in COPD patients followed a median of 7.5 years. Similarly, a recent study (MACRO study of azythromycin), reported an increased all-cause mortality risk for patients that exhibited shorter telomeres (lowest quartile of TL), although this was only observed in the placebo group [19]. COPD as a disease of accelerated aging is associated with earlier mortality. However, the exact mechanism remains unknown but certainly inflammation plays a role, in consequence, some authors propose an “Inflammaging” process [39].

Interestingly, some authors have focused their research on certain molecules and existing drugs in an attempt to unravel how to control telomere attrition. SIRT1, an anti-aging protein, whose activation in mice has been reported to prevent inflammatory responses [40] and to be involved in the reduction of telomeric attrition [41]. On the other hand, telomerase activation has emerged as a potential treatment directed to cases with short telomeres and physiological aging [42]. Recently, metformin, the preferred first-line drug against type-2 diabetes is known to reduce oxidative damage accumulation, chronic inflammation, and increase overall lifespan in mice [43]. Recently, other studies have suggested that metformin use may reduce telomere shortening in adults [44, 45].

This study has several strengths. The most important is its prospective nature (first of its kind) and the excellent phenotypic characterization of the cohort and their outcomes registered annually through 10 years. It is also noteworthy that we were able to calculate the corresponding absolute telomere length data, as supported by the high correlation found between southern blot and the qPCR technique used. However, there are also some limitations: First, telomere length was measured in leucocyte cells and not in lung tissue. However, leukocytes remain the tissue of choice for TL measurement in large cohorts of individuals, because it is accessible and representative of distant tissues [46, 47]. Also, we cannot discard that the shortening of TL differs in different blood cells may vary through time. However, the samples were taken at similar times in all patients, thereby decreasing this potential bias, and TL was measured only if the patients presented blood leukocyte and differential counts values that were within the established normal ranges. Second, although 42 patients out of 263 reached 10 years of observation, their baseline clinical and physiological characteristics were similar to the group as a whole, supporting the validity of the results in these patients as a reflection of COPD as a whole. Furthermore, there were 134 patients having at least two measures of telomere length with a minimum of 6 years follow-up included in the longitudinal analysis (413 observations of clinical and physiological variables) before they were censored or died. Their results provide further support to the conclusions here presented. Also, this is a single center study. A validation cohort would be required; however, this is difficult to achieve due to the complexity of the study design and the time required for monitoring. Another limitation of this study is the absence of histological or imaging data, but this does not detract from the results obtained. Finally, our sample size did not allow us to contrast specific causes of mortality such as cancer or cardiovascular disease, however this does not invalidate the overall findings as the multidimensional nature of the different variables measured moved in the same direction.

## Conclusions

In conclusion, this longitudinal observational study showed that an accelerated telomere shortening over time is associated with worse alveolar gas exchange function, worse lung hyperinflation and extrapulmonary affection in patients with COPD. Moreover, having shorter telomeres is associated with all-cause mortality risk. Studies with larger cohorts with several time points of TL measurements, are needed to validate our findings.

## Supplementary Information

**Additional file 1: Table S1.** Baseline characteristics comparisons between patients with COPD (n = 42) that reached 10 years follow-up vs. the rest of the cohort (n = 221).

**Additional file 2: Figure S1.** Telomere length in patients with COPD distributed by range of age at baseline as follows: ≤ 59 (n = 83), 60–69 (n = 95) and ≥ 70 (n = 85) years old (p = 0.022).

**Additional file 3: Figure S2.** Correlation between telomere length measure by TFR and T/S ratio.

## Data Availability

Data are available upon reasonable request. All data relevant to the study are included in the article or uploaded as additional information.

## Abbreviations

COPD: Chronic obstructive pulmonary disease
T/S: Relative telomere length ratio
TL: Telomere length
qPCR: Quantitative Real Time Polymerase Chain Reaction
CV: Coefficients of variance
T/S_mCh: Change in T/S with respect to its mean over time
HR: Hazard ratio
GLIM: General Linear Modelling for repeated measures test
FEV1: Forced expiratory volume in 1 s
FVC: Forced vital capacity
BMI: Body Mass Index
PaO_2_: Partial oxygen tension
KCO: Transfer factor coefficient of the lung for carbon monoxide, corrected by alveolar volume
IC/TLC: Inspiratory capacity to total lung capacity ratio
6MWD: Six-minute walking distance test
