# Supplementary material for: Telomere length dynamics over 10-years and related outcomes in patients with COPD
Source: Respir Res. 2021 Feb 15;22:56. doi: 10.1186/s12931-021-01616-z (PMC7896411; doi:10.1186/s12931-021-01616-z)
Supplement: Supplementary file 1 — Additional file 1: Table S1. Baseline characteristics comparisons between patients with COPD (n = 42) that reached 10 years follow-up vs. the rest of the cohort (n = 221). [file 12931_2021_1616_MOESM1_ESM.docx]

Supplementary table S1: Baseline characteristics comparisons between patients with COPD (n=42) that reached 10 years follow-up vs. the rest of the cohort (n=221).

| **Variable** | **Patients that did not reach 10 yr follow-up (n=221)** | **Patients with 10 yr follow-up (n=42)** | **p-value** |
| --- | --- | --- | --- |
| Age | 64 ±7 | 61± 8 | **0.023** |
| Sex (male %) | 75 | 67 | 0.256 |
| BMI* | 27±5 | 27±6 | 0.880 |
| Smoking habit (pack-yr)^*†^ | 64±28 | 61±22 | 0.516 |
| Active smoking (%) | 40 | 52 | 0.172 |
| FEV_1_ (L)* | 1.50±0.66 | 1.61±0.61 | 0.322 |
| FEV_1_ (% pred)* | 57±22 | 60±19 | 0.255 |
| FVC (% pred)* | 87±26 | 90±23 | 0.364 |
| FEV_1_/FVC (% pred)* | 51±13 | 54±10 | 0.071 |
| PaO_2_* | 71±12 | 73±10 | 0.266 |
| Kco* | 74±26 | 90±28 | **0.002** |
| IC/TLC (%)* | 34±9 | 35±8 | 0.399 |
| 6MWD (mts)* | 473±101 | 522±84 | **0.004** |
| mMRC dysnea** | 1 (0 - 2) | 1 (0 - 1) | 0.256 |
| BODE index** | 1 (0 - 3) | 1 (0 - 2) | 0.330 |
| Charlson index** | 0 (0 - 1) | 1 (0 - 1) | 0.855 |

*Data are presented as mean ±SD. ** Data are presented as median (25^th^-75^th^pc). BMI: body mass index; FEV_1_: forced expiratory volume in one second; FVC: forced vital capacity; % pred: per cent predicted; PaO_2_: partial oxygen tension; IC/TLC: inspiratory capacity to total lung capacity ratio; SMWD: six minutes walking distance test.
