# Supplementary figures and images for: Telomere length dynamics over 10-years and related outcomes in patients with COPD
Source: Respir Res. 2021 Feb 15;22:56. doi: 10.1186/s12931-021-01616-z (PMC7896411; doi:10.1186/s12931-021-01616-z)

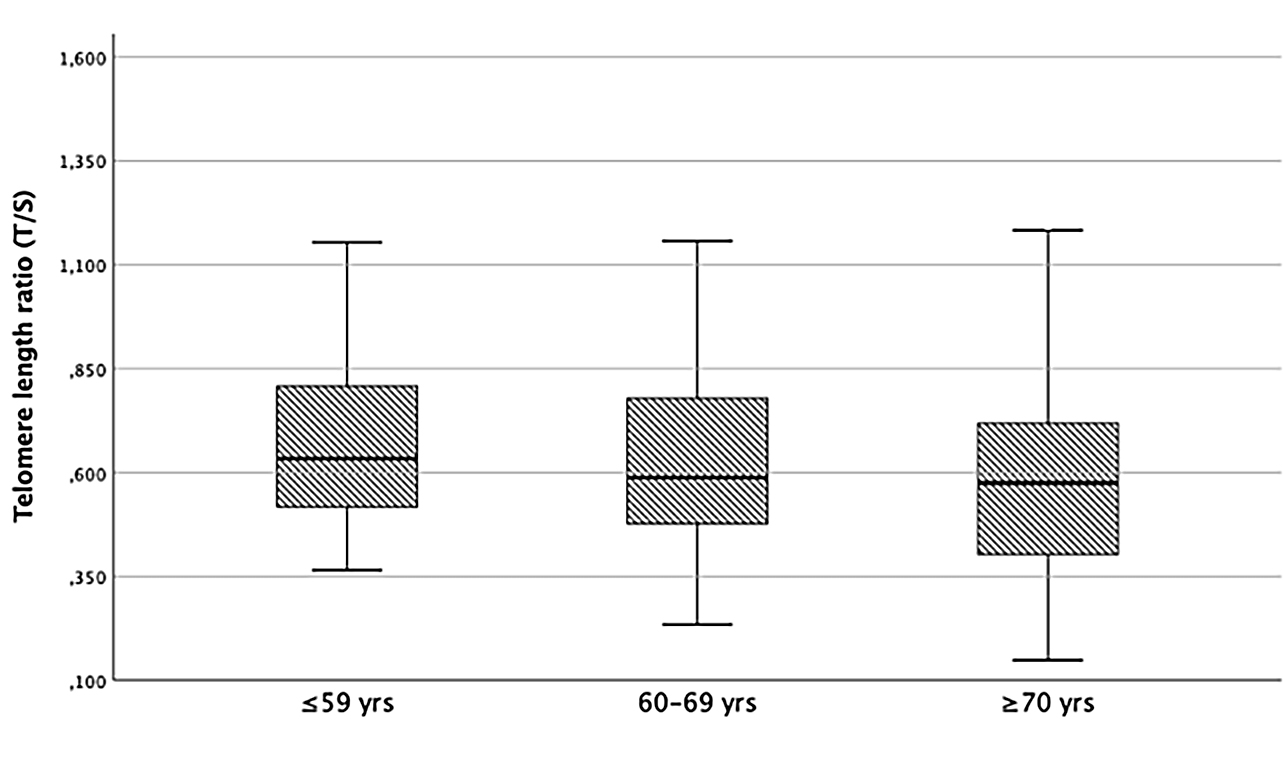

Supplement: Supplementary file 2 — Additional file 2: Figure S1. Telomere length in patients with COPD distributed by range of age at baseline as follows: ≤ 59 (n = 83), 60–69 (n = 95) and ≥ 70 (n = 85) years old (p = 0.022). [file 12931_2021_1616_MOESM2_ESM.jpg]

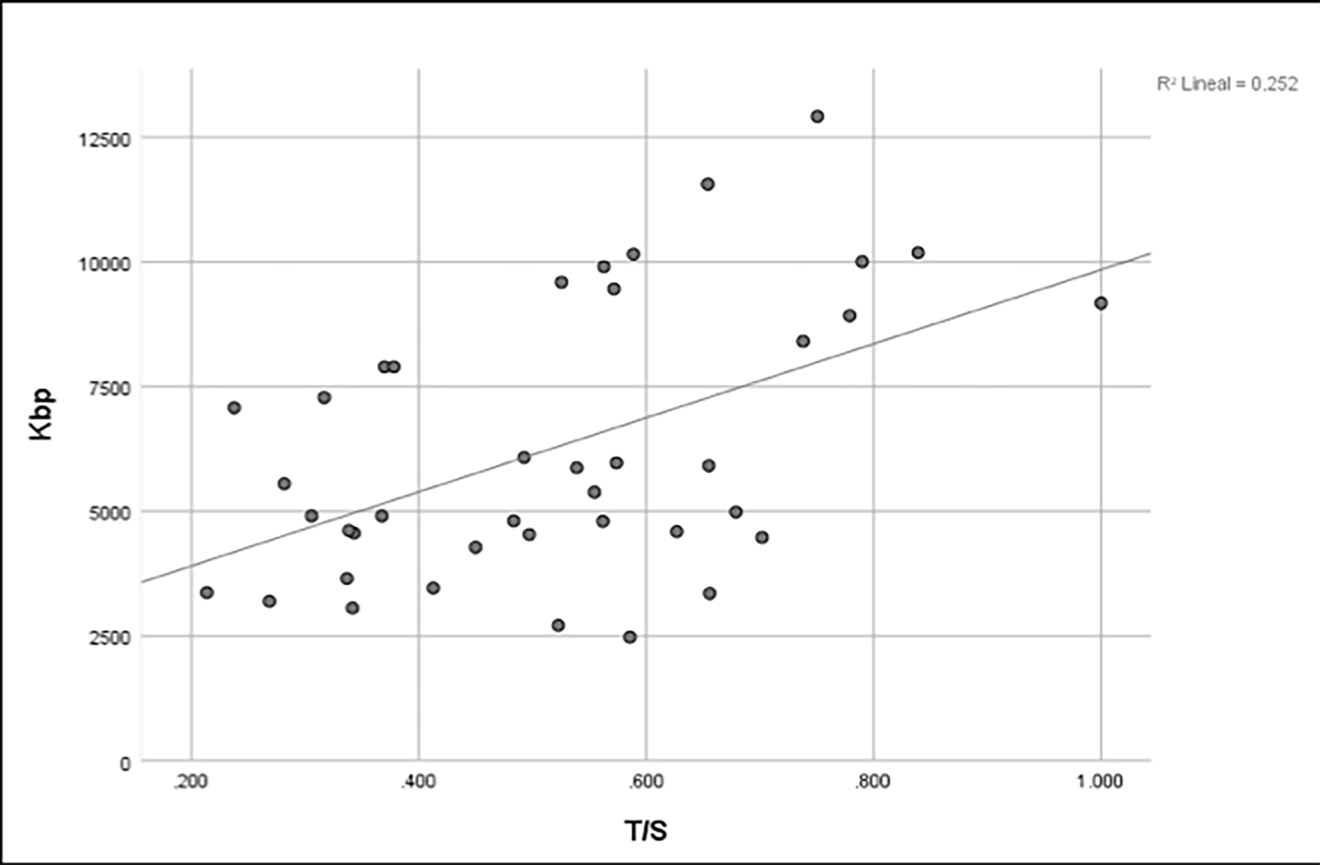

Supplement: Supplementary file 3 — Additional file 3: Figure S2. Correlation between telomere length measure by TFR and T/S ratio. [file 12931_2021_1616_MOESM3_ESM.tif]
